# The Bioeffects Resulting from Prokaryotic Cells and Yeast Being Exposed to an 18 GHz Electromagnetic Field

**DOI:** 10.1371/journal.pone.0158135

**Published:** 2016-07-08

**Authors:** The Hong Phong Nguyen, Vy T. H. Pham, Song Ha Nguyen, Vladimir Baulin, Rodney J. Croft, Brian Phillips, Russell J. Crawford, Elena P. Ivanova

**Affiliations:** 1 Faculty Science, Engineering and Technology, Swinburne University of Technology, PO Box 218, Hawthorn, Vic 3122, Australia; 2 Department d’Enginyeria Quimica, Universitat Rovira I Virgili, 26 Av. dels Paisos Catalans, 43007 Tarragona, Spain; 3 School of Psychology, University of Wollongong, Wollongong, NSW 2522, Australia; Sudbury Regional Hospital, CANADA

## Abstract

The mechanisms by which various biological effects are triggered by exposure to an electromagnetic field are not fully understood and have been the subject of debate. Here, the effects of exposing typical representatives of the major microbial taxa to an 18 GHz microwave electromagnetic field (EMF)were studied. It appeared that the EMF exposure induced cell permeabilisation in all of the bacteria and yeast studied, while the cells remained viable (94% throughout the exposure), independent of the differences in cell membrane fatty acid and phospholipid composition. The resulting cell permeabilisation was confirmed by detection of the uptake of propidium iodine and 23 nm fluorescent silica nanospheres using transmission electron microscopy (TEM) and confocal laser scanning microscopy (CLSM). Upon EMF exposure, the bacterial cell membranes are believed to become permeable through quasi-endocytosis processes. The dosimetry analysis revealed that the EMF threshold level required to induce the uptake of the large (46 nm) nanopsheres was between three and six EMF doses, with a specific absorption rate (SAR) of 3 kW/kg and 5 kW/kg per exposure, respectively, depending on the bacterial taxa being studied. It is suggested that the taxonomic affiliation and lipid composition (e.g. the presence of phosphatidyl-glycerol and/or pentadecanoic fatty acid) may affect the extent of uptake of the large nanospheres (46 nm). Multiple 18 GHz EMF exposures over a one-hour period induced periodic anomalous increases in the cell growth behavior of two *Staphylococcus aureus* strains, namely ATCC 25923 and CIP 65.8^T^.

## Introduction

An electromagnetic field (EMF) is capable of triggering a variety of biological effects [1–4] upon genes [5–9], proteins and enzyme kinetics [10–14], depending on the EMF strength, frequency, and time of interaction [15, 16]. Despite many studies having been undertaken, the mechanisms responsible for the EMF effects are not fully understood and have been the subject of debate [1–4, 8, 10, 12, 16].

Whilst the bulk temperature rises that occur during EMF exposure may impact the cells, several studies have reported specific effects taking place that cannot be explained solely by this increase in bulk temperature. These effects may be a result of microthermal temperature increases that are not detectable at the macro level [4, 15, 17–20], strong polarization effects or subsequent changes in the dielectric constants being induced by the EMF. Other reports, however, suggested that exposure to EMF energy can influence the enzyme kinetics within the cells [15, 17, 21, 22]. Recently, it was reported that exposing bacterial cells to an 18 GHz EMF with a specific energy absorption rate (SAR) of approximately 5.0 kW kg^-1^ at temperature of 40°C induced permeability in the cell walls of *Escherichia coli*, *Staphylococcus aureus*, *Staphylococcus epidermidis*, and *Planococcus maritimus* cells without undermining the viability of the cells [20]. It is thought that the membrane permeation is dependent on the membrane fluidity, which in turn is dependent on the membrane lipid composition, cell microenvironment and the presence of charged phospholipid head groups [23, 24]. Modulation of the membrane fluidity may arise due to the ease of movement of water molecules, and the dielectric constant of water, which is affected by the EMF [25]. It has been reported that a temperature increase would cause an increase in the membrane fluidity, as confirmed by the diffusion of calcein molecules throughout the phosphatidylcholine bilayer membrane [25]. Lande *et al*. (1995) investigated the permeability of various artificial large unilamellar vesicles (LUVs) containing different fatty acid compositions that mimic that of biological membranes [24]. The authors concluded that membrane fluidity was affected by the presence of ionic substances [24]. Bacteria maintained their membrane fluidity by modulating the fatty acid composition in their cell walls [26]. It was also reported that charged phospholipid head groups developed a substantial potential at the lipid ˗ solution interface, influencing the concentration of ions at the interface and hence the permeability properties of the cell membrane [23]. Significant change in the dielectric constant of water due to exposure to the EMF results in the modification of the Debye length, which determines the interaction range between charged groups and thus, can destabilize the lipid bilayer.

Since the fatty acid and phospholipid compositions vary between different bacterial cell types [27, 28], it is of considerable interest to understand whether exposure to 18 GHz EMF will induce cell permeability in typical representatives of the major microbial taxa possessing different compositions of membrane fatty acids and phospholipids (S1 Table). Therefore, the aim of this study was to investigate the effects and dosage requirements of 18 GHz EMF on several prokaryotic organisms that had not been the subject of previous EMF exposure studies, such as *Branhamella catarrhalis* ATCC 23246 (Gram negative bacillus), *Kocuria rosea* CIP 71.15^T^ (Gram positive coccus), *Streptomyces griseus* ATCC 23915 (Gram positive actinobacterium) [29], and the eukaryotic unicellular organism, yeast *Saccharomyces cerevisiae* ATCC 287. The effects resulting from prolonged multiple EMF exposures using two strains of *Staphylococcus aureus* bacteria as model organisms were also studied for the first time.

## Materials and Methods

### Cells, growth conditions and sample preparation

*Branhamella catarrhalis* ATCC 23246, *Kocuria rosea* CIP 71.15^T^, *Staphylococcus aureus* CIP 65.8, ATCC 25923, *Streptomyces griseus* ATCC 23915 bacterial strains, and the yeast *Saccharomyces cerevisiae* ATCC 287 were obtained from the American Type Culture Collection (ATCC, USA), and the Culture Collection of the Pasteur Institute (CIP, France). These cells were selected due to their distinct taxonomic affiliation and differences in their membrane lipid composition and structure. *B*. *catarrhalis* is an aerobic, non-motile Gram-negative diplococcus, opportunistic human pathogen, which is often found in the upper respiratory tract of humans. *B*. *catarrhalis* can cause respiratory infections, acute otitis media, sinusitis and infections such as endocarditis, meningitis and bacterial tracheitis [30]. The bacterium can grow well at temperatures as low as 22°C. Cells are kidney bean shaped, with a diameter of 0.6 to 1.0 μm, often appearing in pairs or as tetrads [31]. *B*. *catarrhalis* is saccharolytic, DNase, oxidase and catalase-positive with butyrate esterase activity [30]. *K*. *rosea* is an aerobic, non-motile, non-encapsulated, non-sporulated Gram-positive coccus, opportunistic human pathogen, which is commonly found on the surface of human skin, mucous membranes, in the oral cavity, and the outer ear canal [29, 32]. The bacterium can also be found in freshwater, saltwater, and soil environments [29, 32]. Cells have a diameter of approximately 1.0 to 1.8 μm and occur in pairs, tetrads or clusters [29]. *Streptomyces griseus* is non-motile, aerobic, Gram-positive filamentous, spore forming rod-shaped bacterium, which is a typical inhabitant of soil [33]. *Streptomyces griseus* is alkaliphilic, produces an aerial mycelium, which has modes of branching that eventually leads the hyphae to form chains of spores called arthospores [33, 34]. The optimum temperature for cell growth is in the range 25 to 35°C [33]. Considered relatively harmless to humans, *Streptomyces griseus* produces many useful secondary metabolites such as enzyme inhibitors, and they comprise 70% of naturally-occurring antibiotics [34]. *Saccharomyces cerevisiae* is considered to be a "model organism" for scientists because it has a fast rate of growth, being both a unicellular and eukaryotic organism [35]. This yeast has been used since ancient times in fermentation processes that convert sugar into alcohol, and baking processes as a leavening agent [35]. Yeast strains can be isolated from the surfaces of plants, surfaces of insects and warm-blooded animals, soils from all regions of the world and even in aquatic environments [35]. The optimum growth temperature is in the range 30 to 35°C [36]. *Saccharomyces cerevisiae* cells are round to ovoid, 5 to 10 μm in diameter, grow in either the haploid or diploid form [36]. Pure cultures were stored at -80°C in nutrient broth (NB, Oxoid Ltd., Basingstoke, Hampshire, England) with the addition of 20% (v/v) glycerol. All strains were grown on nutrient agar (NA, Oxoid Ltd., Basingstoke, Hampshire, England), brain-heart infused agar (BHIA, Becton Dickinson, Sparks, NV, USA) or potato dextrose agar (PDA, Becton Dickinson), depending on the requirements of a particular strain. Prior to each experiment, each strain was grown to the stationary phase of growth as confirmed by growth curves (data not shown) at 25°C (*B*. *catarrhalis*, *K*. *rosea*, *Streptomyces griseus*), 30°C (*Saccharomyces cerevisiae*), or 37°C (*Staphylococcus aureus*). Freshly prepared working suspensions were used for each independent experiment. The bacterial cell density was adjusted to OD_600_ 0.1 in 10 mM phosphate buffered saline (PBS) at pH 7.4, using a spectrophotometer (Dynamica Halo RB-10 UV-Vis, Precisa Gravimetrics AG, Dietikon, Switzerland). The *Streptomyces cerevisiae* yeast cell density was adjusted to 7 × 10^5^ cells mL^-1^ using a Neubauer-improved haemocytometer (Paul Marienfeld, Lauda-Königshofen, Germany).

### EMF exposure

**Fig 1:**
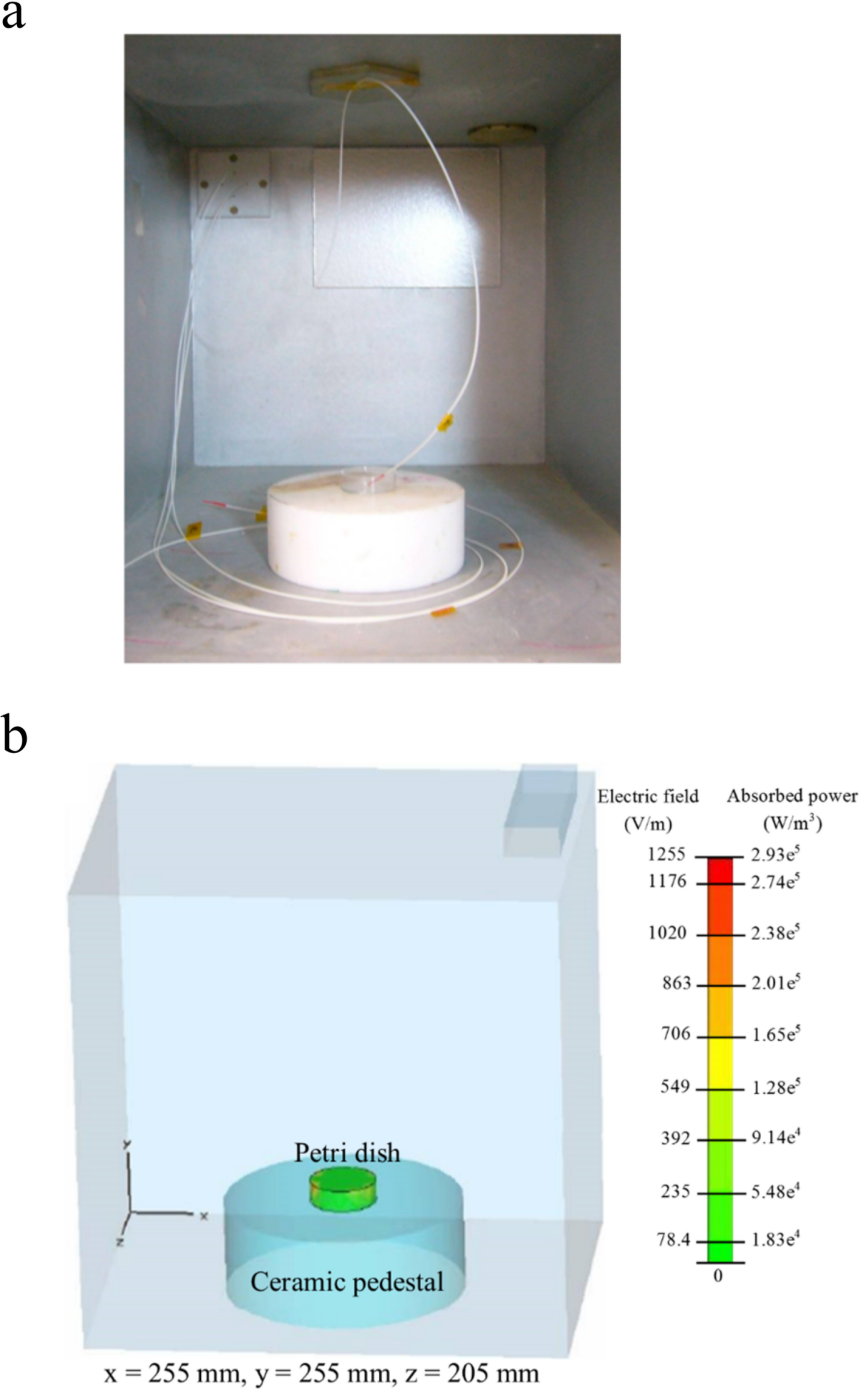
EMF exposure geometry and fraction. **(a)** Configuration of the EMF chamber and **(b)** the electric field and absorbed power were simulated using CST Microwave Studio 3D Electromagnetic Simulation Software.

Exposure of the samples to the EMF was carried out as described elsewhere [4, 20]. In brief, 2 mL of the working suspension was transferred into a micro Petri dish (35 mm diameter, Griener Bio One, Frickenhausen, Germany). The EMF apparatus used for all experiments was a Vari-Wave Model LT 1500 (Lambda Technologies, Morrisville, NC, USA) instrument with a fixed frequency of 18 GHz. The samples were placed onto a ceramic pedestal PD160 (Pacific Ceramics, Sunnyvale, CA, USA, ε’ = 160, loss tangent < 10^−3^) at a position that had been identified, using electric field modelling of CST Microwave Studio 3D Electromagnetic Simulation Software (CST MWS) (CST of America, Framingham, MA, USA), to be the position that provided the most consistent heating environment (Fig 1). The calculated wavelength of the EMF in water was determined to be 2.34 mm, which is greater than the linear dimensions of each bacterial cell. The depth of penetration was calculated to be 1.04 mm, which was greater than the thickness of the bacterial suspension in the Petri dish. Hence, the possibility of subjecting the samples to non-even heating due to the presence of a non-uniform field distribution was considered to be negligible. The temperature of the suspension was constantly monitored during EMF exposures via a built-in temperature probe, a Luxtron Fiber Optic Temperature Unit (LFOTU) (LumaSense Technologies, Santa Clara, CA, USA), and a portable Cyclopes 330S infrared/thermal monitoring camera (Minolta, Osaka, Japan).

### Bulk heat treatment

A Peltier plate heating/cooling system (TA Instruments, New Castle, DE, USA) was used to replicate the bulk temperature profiles achieved during the EMF exposure experiments. All Peltier plate heated samples were performed in parallel with the EMF exposure experiments. At least three independent experiments were conducted. Working suspensions that were not subjected to either EMF exposure or Peltier plate heating were used as the negative controls for all experiments.

### Cell treatment and viability assays

*B*. *catarrhalis*, *K*. *rosea*, *Streptomyces griseus*, and *Saccharomyces cerevisiae* cells were subjected to three consecutive EMF exposures with SAR doses of approximately 5.0 kW kg^-1^ that resulted in a temperature increase ranging from 20°C to 40°C (at a heating rate of 20°C per min) for 1 min, then the sample was allowed to cool to 20°C on ice (at a rate of 10°C per min) between exposures. The liquid evaporation that took place during the EMF exposures was found to be approximately 1.5%, determined using an analytical balance (Cheetah Scientific, France). This loss of mass was regarded as being negligible.

The counting of viable cells was conducted immediately after EMF exposures and in parallel with those of the control samples by plating 100 μL cell suspensions on the agar plates containing solidified nutrient media: nutrient agar (NA, Oxoid Ltd., Basingstoke, Hampshire, England), brain-heart infused agar (BHIA, Becton Dickinson, Sparks, NV, USA) or potato dextrose agar (PDA, Becton Dickinson), depending on the requirements of a particular strain. Ten plates for each bacterial strain were used. Three technical replicates were carried out to allow a statistical analysis of the results to be performed. Three independent Student t-tests were performed to compare the consistency of the cell viability rates for each set of experimental conditions, exposure trials and test strains.

### Confocal Laser Scanning Microscopy (CLSM) analysis

Propidium iodide (1.0 mg mL^-1^ solution in water; Life Technologies Australia, Mulgrave, Australia) was used at a concentration of 500 nM to directly determine the permeability of the cells being studied [37]. Non-treated cells, cells inactivated by boiling (100°C) and Peltier plate heat-treated cells were used as three different types of control. Heat inactivated cells were prepared by boiling the cell suspension for 15 min, followed by cooling in a 25°C water bath for 30 min. PI was added to all the cell suspensions at 1 min and 10 min after EMF exposures. The PI remained in contact with the studied cells throughout the experiment.

Two types of fluorescent, hydrophilic, neutrally charged silica nanospheres, 23.5 ± 0.2 nm (FITC) and 46.3 ± 0.2 nm (Rhodamine B) (Corpuscular, Cold Spring, NY, USA) were used. These silica nanospheres were selected because hydrophilic nanoparticles rarely translocate through a lipid bilayer [38] and neutrally charged nanoparticle surfaces prevent any nonspecific interactions taking place with the membrane [39]. The nanospheres were added to the cell suspensions 1 min after the EMF exposure at a concentration of 25 μg mL^-1^, incubated for 10 min then washed twice by centrifugation at 4500 rpm for 5 min. A 100 μL aliquot of each sample was then analyzed using a Fluoview FV10i-W inverted microscope (Olympus, Tokyo, Japan). Approximately 10 CLSM images per EMF exposure group were obtained.

### EMF permeability coefficient

The nanosphere loading capacity of the EMF exposed bacterial and yeast cells were quantified according to the fluorescence intensity of any silica nanospheres that were taken into the cells. A POLARstar Omega microplate reader (BMG Labtech, Ortenberg, Germany) was used to measure the fluorescence intensity of nanospheres in each cell suspension. Each sample was prepared according to the method used for CLSM analysis. A calibration curve was constructed to determine the correlation of fluorescent intensity and nanosphere concentration. A total of seven nanosphere concentrations were prepared (0.005, 0.05, 0.5, 1, 5, 19 and 15 μg mL^-1^).

The mass *m* of a silica nanosphere was determined from the mass density of silica *ρ* and is the volume of a silica nanosphere *V*, related to the radius *r as* $V=\frac{4}{3}\pi r^{3}$.

The radii of the 23.5 nm and 46.3 nm nanospheres were 11.75 × 10^−7^ cm and 23.15 × 10^−7^ cm, (Corpuscular), volume 6.8 × 10^−18^ and 5.2 × 10^−17^ cm^3^, and mass 1.8 × 10^−17^ and 1.38 × 10^−16^ g, respectively. The mass of a single nanosphere was used to calculate the number of internalized nanospheres.

### EMF permeability dosimetry

The number of viable cells on agar plates was evaluated after subsequent EMF exposures to confirm that at least 85% of the cells survived the exposure under these conditions. For the bacteria that did not survive, the maximum temperature was decreased to 33°C (with a heating rate of 13°C per min for 1 min), allowing the sample to cool to 20°C on ice for 1.3 min (at a rate of 10°C per min) between exposures. The CLSM analysis of the samples was then carried out to study the permeability of the cells. If no uptake of nanospheres was observed, the number of EMF exposures was increased to four, five or six consecutive exposures. SEM was also used to monitor any changes in cell morphology.

The SAR was calculated with the assumption that all the absorbed field energy was transformed into heat, and disregarding any heat dissipation, as:

$$\text{SAR}=\text{c}\times \frac{\partial \text{T}}{\partial \text{t}}|\text{t}=0$$ (1)

where *c* is the specific heat capacity of the medium (kJ kg^-1^°C^-1^), and $\frac{\partial T}{\partial t}|t=0$ is the time derivative of the temperature determined at t = 0 (°C s^-1^).

The SAR of each EMF exposure was calculated using Eq (1) by measuring the temperature change over time under the optimum settings (power, temperature and number of treatments). It was assumed that the specific heat capacity of the cell suspension was the same as that of water at 25°C, which is 4.18 kJ kg^-1^°C^-1^. The SAR was determined experimentally in this work because it has been suggested that it is a more accurate estimation of energy absorption for biological material [40–42]. This is because any variation in the specific heat within a sample of biological matter is usually much smaller than the corresponding variation in conductivity, resulting in a much more uniform temperature than determined using an electric field distribution [40–42].

### Scanning Electron Microscopy (SEM) analysis

A field emission scanning electron microscope FeSEM–SUPRA 40VP (Carl Zeiss, Jena, Germany) with a primary beam energy of 3 kV was used to obtain high-resolution images of the cell samples. A 100 μL aliquot of EMF-exposed suspension was placed on a glass cover slip (ProSciTech, Kirwan, Australia) in duplicate for each EMF exposure experiment. After 20 min, the glass cover slips were washed with nanopure H_2_O (with a resistivity of 18.2 MΩ cm^-1^), dried with 99.99% purity nitrogen gas, then subjected to gold sputtering (6 nm thick gold film) using a NeoCoater MP-19020NCTR (JEOL, Frenchs Forest, Australia). Approximately ten SEM images were obtained at 5,000× (yeast) and 70,000× (bacteria) magnifications for subsequent analysis.

### Transmission Electron Microscopy (TEM)

The cell suspensions subjected to MW exposure and with the addition of the 23.5 nm nanospheres were pelleted by centrifugation at 4800 rpm for 5 min at 25°C. The cells were then washed twice with phosphate buffer saline (PBS) in order to remove any unbound nanospheres. The pellets were then suspended in 2 mL of 4% glutaraldehyde in PBS (10 mM, pH 7.4) for 30 min, and washed twice in PBS for 5 min. After the final washing step, the cells were mixed thoroughly in 0.5 mL of 5% agarose gel by stirring. The agar was immediately cooled to 4°C by refrigeration for 30 min, then cut into 1 mm^3^ cubes and fixed with 1 mL of 1% osmium tetroxide (OsO_4_) for 1 h. The cubes were washed twice in nanopure H_2_O (with resistivity of 18.2 MΩ cm^-1^) for 15 min. The cells were dehydrated by passing the cubes through a graded ethanol series (20%, 40%, and 60%) (2 mL) for 15 min and stained for 8 h with 2% uranyl acetate in 70% ethanol (2 mL). After staining, the cells were further dehydrated by passing the cubes through another graded ethanol series (80%, 90% and 100%) for 15 min (2 mL) [43, 44].

The embedding medium was prepared using Araldite, dodecenyl succinic anhydride (DDSA), and benzyldimethylamine (BDMA) (ProSciTech) and stirred thoroughly [45]. In order to embed the samples, each cube was washed twice with 100% acetone (2 mL) for 20 min, then incubated in 2 mL of acetone and embedding medium (1:1 ratio) for 8 h, followed by transfer to acetone and embedding medium (1:3 ratio) for 8 h and finally transferred into the pure embedding medium for 8 h. The cube was then transferred into an embedding mold containing fresh pure embedding medium, which was then polymerized for 24 h at 60°C [46]. The final block was trimmed, then cut into ultrathin sections (80 nm thickness) using a Leica EM UC7 ultramicrotome (Leica Microsystems, Wetzlar, Germany) with a glass knife. Sections were placed onto 200 mesh copper grids and examined using a JEM 1010 instrument (JEOL).

### Multiple EMF exposure experiments

Working suspensions of two strains, *Staphylococcus aureus* ATCC 25923 and *Staphylococcus aureus* CIP 65.8^T^, were divided into eighteen batches, each of 2 mL volume. Eighteen batches of samples were subjected to a series of EMF exposures ranging in frequency from 1 to 18 GHz. The first batch was exposed to one EMF dose, which resulted in a temperature increase in the samples ranging from 20°C to 40°C (at a heating rate of 20°C per min) for 1 min. Samples were then allowed to cool to 20°C on ice (at a rate of 10°C per min). The second batch was exposed to two consecutive EMF doses, and so on. The working cell suspensions, which were left untreated for the same period of time as those of the EMF exposed samples, were used as untreated controls. All the cell viability tests were conducted immediately after EMF exposure experiments and in parallel with the untreated control. The number of viable bacteria as a function of the number of EMF exposures was identified by a one-way analysis of variance (ANOVA) using the general linear model procedure. Mean values were separated by Tukey’s Honestly Significant Differences test (*p* < 0.05). All statistical analyses were completed using SPSS v.21 (SPSS Inc., Chicago, IL, USA).

## Results and Discussion

### EMF-induced cell permeability

**Fig 2:**
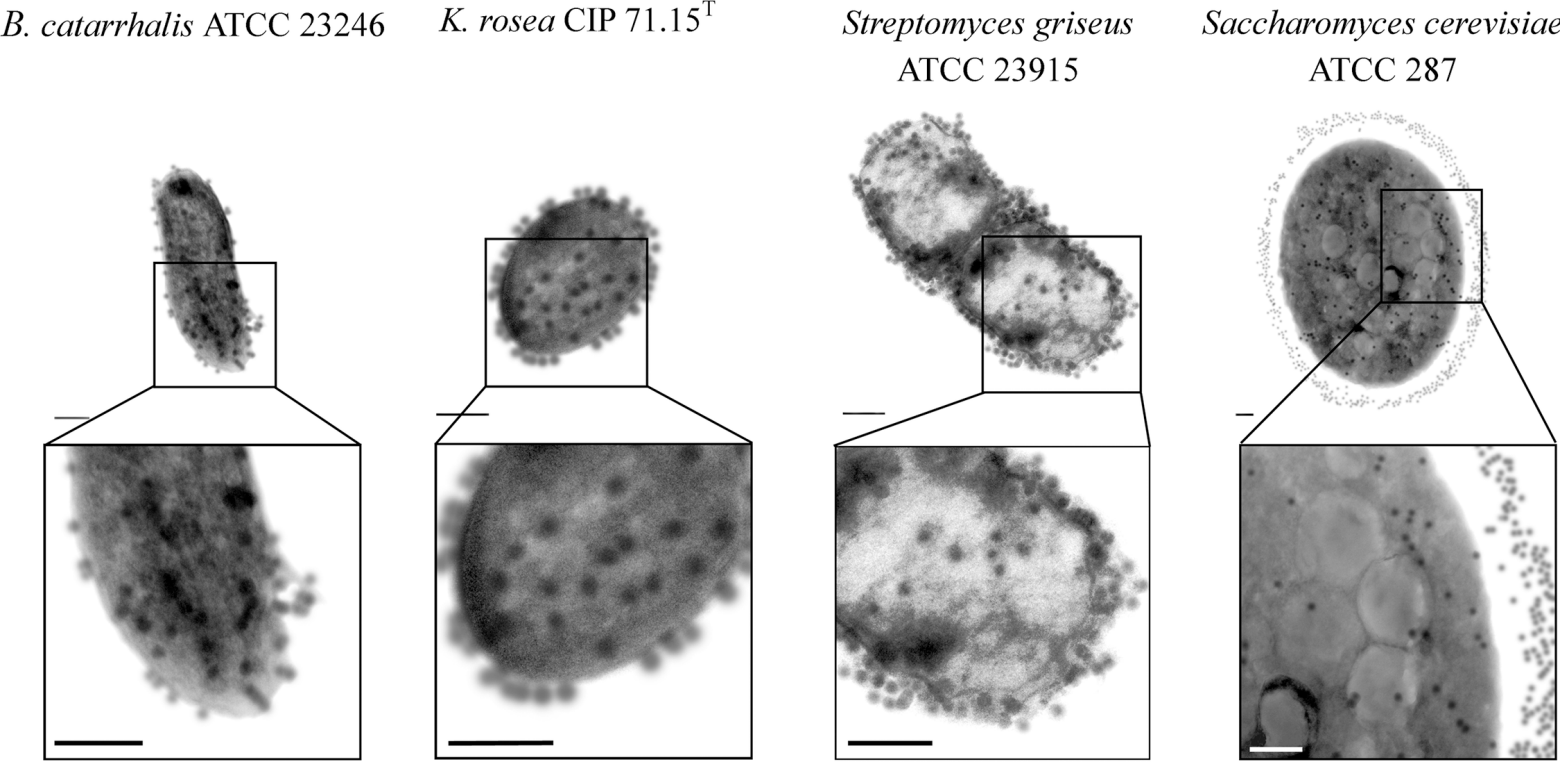
Internalisation of 23.5 nm nanospheres into the EMF exposed cells. Typical TEM images of ultra-thin (80 nm) cross-sections of *B*. *catarrhalis*, *K*. *rosea*, *Streptomyces griseus* and *Saccharomyces cerevisiae* cells showing the uptake of 23.5 nm nanospheres. The number of nanospheres around the Gram positive bacterial cells and yeast cells were found to be considerably greater than that found around the Gram negative bacterial cells (Indicated by arrows). Scale bars are 200 nm.

**Fig 3:**
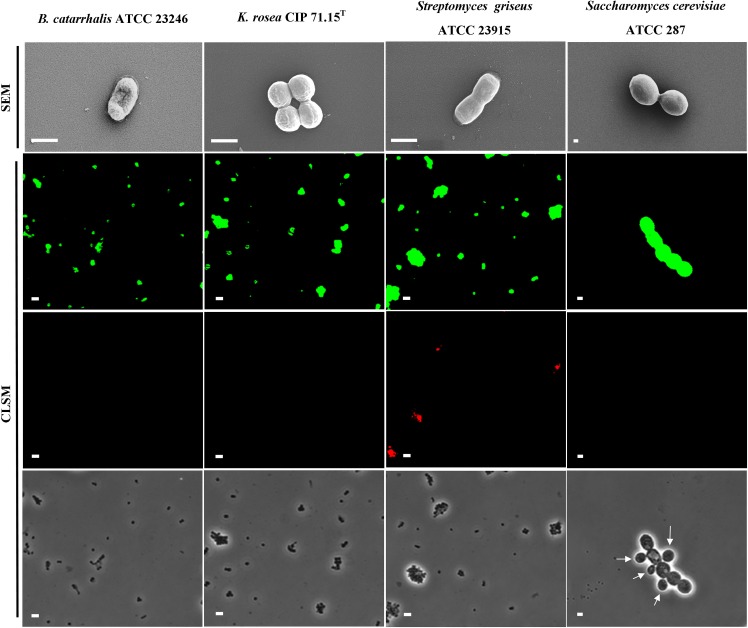
The effects of 18 GHz EMF radiation on the cells under investigation. Typical SEM micrographs of *B*. *catarrhalis*, *K*. *rosea*, *Streptomyces griseus* bacteria and *Saccharomyces cerevisiae* yeast cells after exposure to 18 GHz EMF radiation. No significant change in cell morphology was observed. Scale bars 1 μm (top row). CLSM images show an uptake of the 23.5 nm nanospheres (second row) and 46.3 nm nanospheres (third row), after cell walls were permeabilised as a result of EMF exposures. The phase contrast images (in the bottom row) show the cells in the same field. Arrow indicates young yeast cells, which were unable to uptake any of the nanosheres. Scale bars 5 μm (second, third and bottom rows).

**Fig 4:**
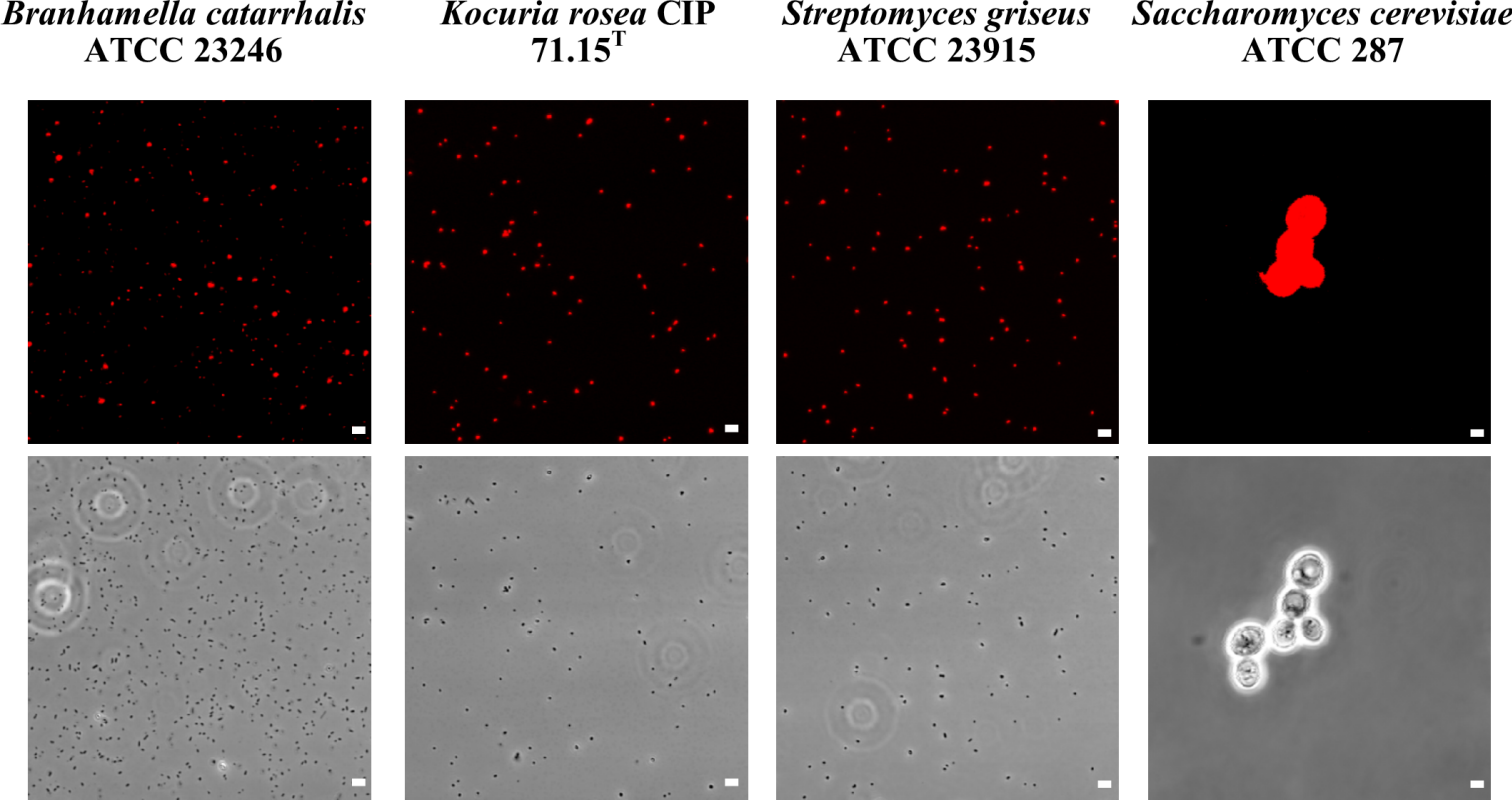
Propidium iodide internalization by the studied cells after EMF exposure. CLSM images showing propidium iodide internalization after EMF exposure (first row). Phase contrast micrographs showing cells in the same field of view (second row). Scale bars in all fluorescence images are 5 μm.

In order to investigate the effect of the 18 GHz EMF exposure on the bacterial and yeast cells, the uptake of two sizes of fluorescent, hydrophilic, non-charged silica nanospheres of 23.5 nm and 46.3 nm was measured. Analysis of the TEM micrographs (Fig 2 and S1 Fig) and CLSM imaging (Fig 3 and S2 Fig) confirmed the uptake of 23.5 nm nanospheres in all studied organisms (up to 97% permeabilised cells). The uptake of propidium iodide (Fig 4 and S3 Fig) also reconfirmed that the EMF induced membrane permeability of the studied cells. A statistical analysis of the data did revealed that there was no statistically significant difference between the Peltier heated and untreated cells (*B*. *catarrhalis* (*p* > 0.05), *K*. *rosea* (*p* > 0.05), *Streptomyces griseus* (*p* > 0.05) and *Saccharomyces cerevisiae* (*p* > 0.05)). There were, however, statistically significant differences between the EMF exposed cells and the control samples (p < 0.05).

This work indicated that three 18 GHz EMF exposures with 1 min SAR doses of approximately 5.0 kW kg^-1^ (at 40°C) consistently induced cell wall permeabilisation in all of the studied bacterial cells and yeast, regardless of the differences in their cell wall/ membrane structures. A TEM analysis of the ultra-thin (90 nm) cross-sections of the cells confirmed the intracellular location of the 23.5 nm nanospheres. Although the nanospheres were observed entering into the cells themselves, they were also found to be present on the surface of the cell walls (Fig 2). It was observed that the number of nanospheres around the Gram positive bacterial cells and yeast were considerably more than that observed around the Gram negative bacterial cells. It could be speculated that the peptidoglycan layer of the Gram positive bacterial cell walls and mannoprotein/β-glucan layer of the yeast cell wall [47] may have played a role in filtering and/or trapping the nanospheres. Control groups of the untreated and Peltier plate heated cells were found not to have taken up any of the nanospheres (S1 and S2 Figs), highlighting the lack of cell permeability in these samples.

Some bacteria such as *B*. *catarrhalis* and yeast were not able to take up the larger (46.3 nm) nanospheres. Although the larger nanospheres were detected in the *K*. *rosea* and *Saccharomyces cerevisiae* cells, the viability of these organisms under these SAR doses remained low (62% and 47%, respectively, as shown in Table 1), and therefore the EMF dosages for these bacteria were further optimized (see next section).

**Table 1:** Dosimetry and cell viability after 18 GHZ EMF exposure.

Cell types	Taxonomy affiliation	Dosimetry		Cell viability	
		Doses	SAR (kW/kg)	33°C	40°C
*Branhamella catarrhalis* ATCC 23246*	*Bacteria; Proteobacteria; Gammaproteobacteria; Pseudomonadales; Moraxellaceae; Moraxella; Branhamella*	3	5	N/A	96% ± 7%
*Escherichia coli* K 12	*Bacteria; Proteobacteria; Gammaproteobacteria; Enterobacteriales; Enterobacteriaceae; Escherichia*	3	5	N/A	88% ± 4%
*Kocuria rosea* CIP 71.15^T^*	*Bacteria; Actinobacteria; Actinobacteria; Actinobacteridae; Micrococcales; Micrococcaceae; Kocuria*	3	3	98% ± 7%	62% ± 10%
*Planococcus maritimus* KMM 3738	*Bacteria; Firmicutes; Bacilli; Bacillales; Planococcaceae; Planococcus*	3	5	N/A	85% ± 8%
*Staphylococcus aureus* ATCC 25923	*Bacteria; Firmicutes; Bacilli; Bacillales; Staphylococcaceae; Staphylococcus*	3	5	N/A	85% ± 5%
*Staphylococcus aureus* CIP 65.8^T^	*Bacteria; Firmicutes; Bacilli; Bacillales; Staphylococcaceae; Staphylococcus*	3	5	N/A	89% ± 5%
*Staphylococcus epidermidis* ATCC 14990^T^	*Bacteria; Firmicutes; Bacilli; Bacillales; Staphylococcaceae; Staphylococcus*	3	5	N/A	84% ± 9%
*Streptomyces griseus* ATCC 23915*	*Bacteria; Actinobacteria; Actinobacteria; Actinobacteridae; Streptomycetales; Streptomycetaceae; Streptomyces; Streptomyces griseus group; Streptomyces griseus subgroup*	3	5	N/A	97% ± 7%
*Saccharomyces cerevisiae* ATCC 287*	*Eukaryota; Opisthokonta; Fungi; Dikarya; Ascomycota; saccharomyceta; Saccharomycotina; Saccharomycetes; Saccharomycetales; Saccharomycetaceae; Saccharomyces*	6	3	94% ± 8%	47% ± 8%

*Data from this study.

The cell viability, expressed as a percentage, was calculated by direct counting the colony forming units present on the plates. The data are mean values, with errors being the standard deviation (SD). Results are representative of 3 independent experiments. N/A: not available. The control samples were subjected to conventional heating using a Peltier plate method; this heating method resulted in approximately 95% viability.

**Fig 5:**
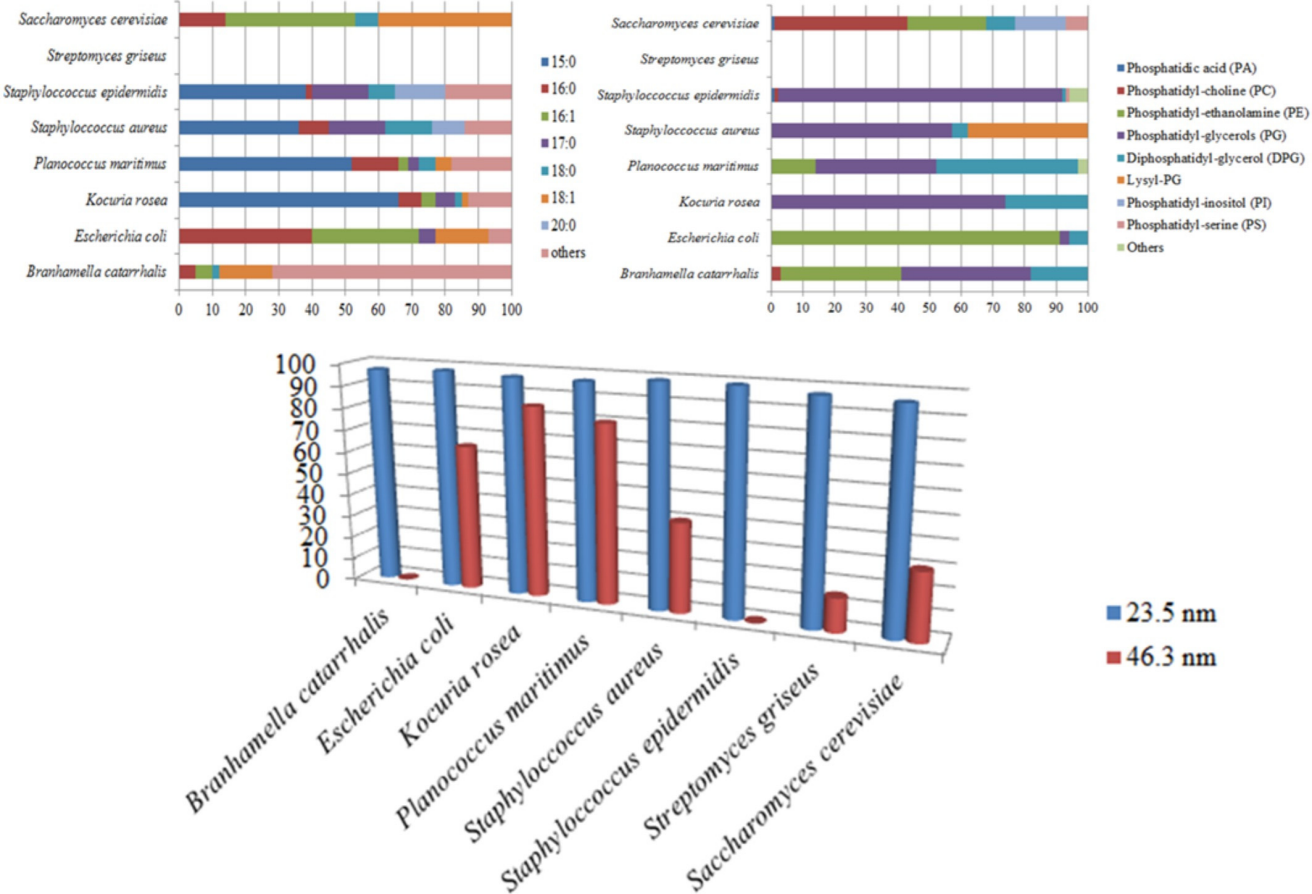
Lipid composition of cell membranes and cell permeability under 18 GHz EMF exposure (3 doses of 5 kW/kg). All cell types were consistently permeabilized, allowing the uptake of 23.5 nm nanospheres. The amount of phospholipid headgroup PG and C 15:0 fatty acid in cell membrane seems to be critical in determining the ability of the cells to internalize the large nanospheres (46.3 nm). In the above sub-figures, the x-axis represents the percentage of total lipid occurrence and the y-axis represents the binomial names and type of lipids (fatty acid tails in the above left sub-figure and phospholipid headgroups in the above right sub-figure). In the below sub-figure, the y-axis represents the percentage of cells that are able to internalize the nanospheres.

It should be noted that it was previously reported that under the same EMF exposure conditions, *Staphylococcus epidermidis* cells were not able to take up the 46.3 nm-nanospheres, yet *Staphylococcus aureus* cells were able to do so [20]. A comparative analysis of the phospholipid and fatty acid compositions (Fig 5) indicated that *Staphylococcus epidermidis* cells have greater proportion of phosphatidyl-glycerol (PG) and less lysyl-PG compared to those levels reported for *Staphylococcus aureus* [48]. It is likely that the PG head-groups contributed to the membrane fluidity, affecting the uptake of the 46 nm nanospheres. Some variation in the uptake of the large nanospheres was noted for the Gram-positive *B*. *catarrhalis* and Gram-negative *E*. *coli* bacterial cells. For example, a substantial proportion of PG has been reported to be present in the *B*. *catarrhalis* membrane phospholipids; this higher proportion of PG did not allow the internalisation of 46.3 nm nanospheres. The *E*. *coli* cell membranes, however, contained a greater proportion of phosphatidyl-ethanolamine (PE) and were able to internalise the 46 nm nanospheres [20]. It is important to note that other components of the cell membrane may also play a role in the permeabilisation of the cell membrane. For example, it appeared that although PG comprises up to 30% of the membrane phospholipids in *P*. *maritimus* cells, this bacterium was found to be able to internalise a majority of the 46.3 nm nanospheres. Amongst the strains tested, it is likely that the greater proportion (up to 65% of total fatty acids) of 15:0 fatty acid, which has the lowest melting temperature (33.5°C, S1 Table), might increase the susceptibility of *P*. *maritimus* cells to the EMF exposure. Hence, it is suggested that the amount of the phospholipid head-group (PG) and C 15:0 saturated fatty acid present in the cell membrane may play an important role in the destabilisation of lipid bilayer of the membrane by EMF exposure, such that the cells are then able to internalise the 46 nm nanospheres.

It is also believed that the type and extent of these membrane effects would be strongly dependent on the EMF frequencies being used, as higher frequency fields affect smaller molecules to a greater extent [49]. While oscillations of EMF in the GHz frequency range are too fast to move larger molecules such as proteins and other biomolecules, they can induce the rotation of water dipoles [50]. These rotations and reorientations of dipoles are responsible for the energy loss in consequent heating induced by the EMF. The 18 GHz EMF acts directly on water molecules inducing their rotation and reorientation with polarization lag responsible for dissipation of energy and heating. Larger molecules should not be affected by such rapidly alternating fields, since their rotational frequencies are sufficiently lower, while vibrational frequencies of individual groups have low energy compared to kinetic energy [49]. The rotation of water molecules disrupts hydrogen bonding network and affects the water properties. As a result, dielectric constant of water, *ε*, is decreased with frequency *ϖ* according to:

$$\varepsilon (\varpi )=\frac{\varepsilon_{s}-\varepsilon_{\infty }}{1+\varpi^{2}\tau^{2}}+\varepsilon_{\infty }$$ (2)

where *ε*_*s*_ and *ε*_∞_ are the dielectric constants of water at zero (static) and infinite (optical limit) frequencies. The relaxation time of water molecule $\tau =\frac{4\pi \eta a^{3}}{kT}$ depends on temperature *T*, the radius of the molecule *a* and the viscosity *η*.

It was reported that the maximum EMF absorption by water at 20°C and 40°C occurred at 18 GHz [51] and 25 GHz [52], respectively. Apart from heating of water, the decreased dielectric constant affected the interaction between charges and Debye (screening) length, which is a measure of the screening of the electrostatic interactions in polarizable and salty media. In the absence of EMF, the Debye length is given by:

$$\lambda_{D}=\sqrt{\frac{\varepsilon_{s}kT}{4\pi e^{2}\rho }}$$ (3)

where *e* is the elementary charge and *ρ* is the density of counterions and salt molecules.

The change in the dielectric constant affects the electrostatic interaction between charges in water media and thus, it can affect the stability of charged bilayers composed of polar lipids. This, together with the heating, are believed to change the physicochemical state (*i*.*e*., fluidity) of the cell membranes and could render them more sensitive to membrane deformation [24, 28, 53]. This modulation of the mechanical stimulation in turn changes the membrane tension, causing it to deform, resulting in an enhanced degree of membrane trafficking via exocytosis/endocytosis, as was previously reported for several bacterial cell types [20, 54, 55].

Other studies have suggested that the EMF-induced changes in water properties can alter the conformation of proteins, their degree of hydration, and other properties that results a change in their activity [22, 56–59]. It was demonstrated that exposing bacteria to an EMF of 51.8, 53, 70.6, 73 and 90 GHz frequency resulted in changes in the enzymatic activities and ion (H^+^ and K^+^) transport processes through the plasma membrane [56–59].

The corresponding concentration of 23.5 nm nanospheres as a function of relative fluorescence units (RFU) was found to be approximately 0.33 μg mL^-1^ for *B*. *catarrhalis*, 0.25 μg mL^-1^ for *K*. *rosea*, 0.36 μg mL^-1^ for *Streptomyces griseus* and 0.35 μg mL^-1^ for *Saccharomyces cerevisiae* cells. By dividing the concentration of the nanospheres by the total cell concentration in the cell suspension (10^8^ bacterial cells mL^-1^ and 7 × 10^5^ yeast cells mL^-1^), the mass of the internalized nanospheres was found to be approximately 3.3 fg cell^-1^ (*B*. *catarrhalis*), 2.5 fg cell^-1^ (*K*. *rosea*), 3.6 fg cell^-1^ (*Streptomyces griseus*) and 50 pg cell^-1^ (*Saccharomyces cerevisiae*). Divided by the mass of a single 23.5 nm nanosphere, the number of internalized nanospheres per single bacterium was 183 spheres cell^-1^ (*B*. *catarrhalis*), 139 spheres cell^-1^ (*K*. *rosea*), 200 spheres cell^-1^ (*Streptomyces griseus*) and 2.8 × 10^4^ spheres cell^-1^ (*Saccharomyces cerevisiae*). The number of 46.3 nm nanospheres that were internalized by the bacteria was similarly quantified (Table 2).

**Table 2:** Internalization of silica nanospheres by bacterial and yeast cells after EMF exposures.

	Silica nanospheres			
	23.5 nm		46.3 nm	
Bacterial strains	Loading capacity *	Amount of cells took up nanospheres(%)	Loading capacity *	Amount of cells took up nanospheres (%)
*Branhamella catarrhalis* ATCC 23246*	183 ± 8	98 ± 4	Not detected	Not applicable
*Kocuria rosea* CIP 71.15^T^*	139 ± 8	99 ± 5	62 ± 8	83 ± 8
*Planococcus maritimus* KMM 3738	172 ± 8	97 ± 5	75 ± 8	80 ± 9
*Staphylococcus aureus* ATCC 25923	161 ± 8	99 ± 4	81 ± 8	40 ± 7
*Staphylococcus aureus* CIP 65.8^T^	261 ± 8	99 ± 3	114 ± 8	44 ± 7
*Staphylococcus epidermidis* ATCC 14990^T^	211 ± 8	99 ± 5	Not detected	Not applicable
*Streptomyces griseus* ATCC 23915*	200 ± 8	99 ± 5	109 ± 8	55 ± 8
*Saccharomyces cerevisiae* ATCC 287*	27778 ± 8	97 ± 5	Not detected	Not applicable

* per single cell

Nanosphere loading capacity was calculated using the fluorescence intensity of nanospheres. The number of bacterial and yeast cells that were able to internalize the nanospheres, expressed as a percentage, was calculated by counting fluorescent cells in the CLSM images. Data are means ± SD and are representative of 3 independent experiments.

### EMF permeability dosage requirements

Cell viability experiments and CLSM analysis of the EMF exposed *K*. *rosea* and *Saccharomyces cerevisiae* cells revealed that three and six exposures with 1 min SAR doses at of approximately 3.0 kW kg^-1^ (at 33°C) were sufficient to induce cell permeability of the viable cells. In the case of *B*. *catarrhalis* and *Streptomyces griseus* cells, only three exposure doses at 40°C were required to induce membrane permeability (Table 1, Figs 1 and 2). This dose specific permeabilisation implies that 18 GHz EMF can be used to target a specific type of cells and hence a new drug delivery/release method. The lack of permeabilisation of young yeast cells (Fig 3) was observed, however due to the lack of data pertaining to the composition of the young yeast cell wall, the reason of this phenomenon remains unclear. Certain biological effects were previously attributed to the EMF of SAR dose of 4.0 kW kg^-1^ at 8.53 GHz, 4.85 kW kg^-1^ at 2.45 GHz, *e*.*g*., three-dimensional conformational changes in green fluorescent protein (GFP) [60] and increased citrate synthase binding efficiency [10].

### Effect of multiple 18 GHz EMF exposures on *Staphylococcus aureus* strains

**Fig 6:**
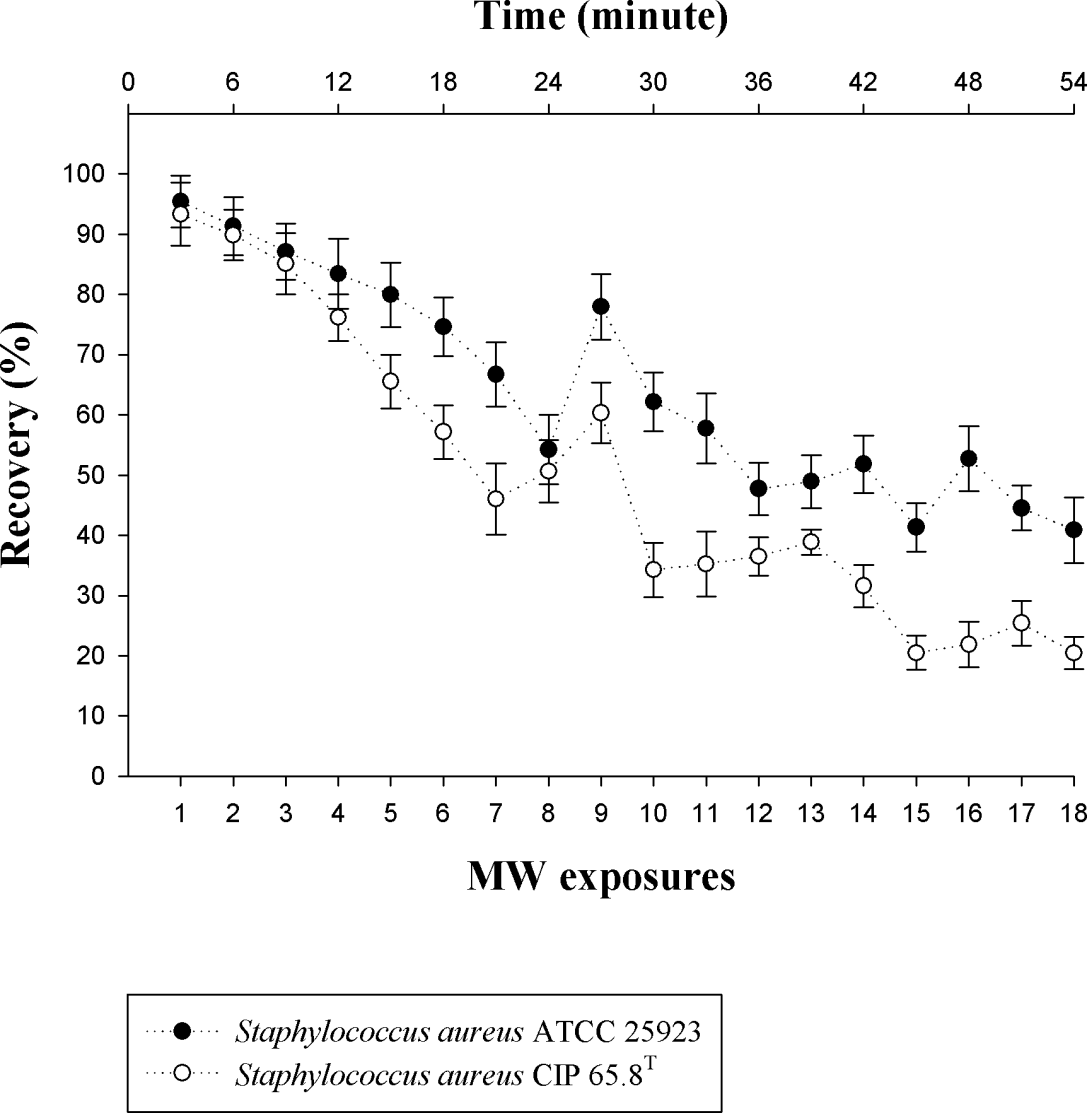
The effect of multiple 18 GHz EMF exposures on the viability of *Staphylococcus aureus* cells. *Staphylococcus aureus* ATCC 25923 (shaded white) and *Staphylococcus aureus* CIP 65.8^T^ (dotted grey) cell viability as a function of time and number of EMF exposures. The cell viability of the two *Staphylococcus aureus* strains is displayed in colony forming units (cfu) per 100 μL. The untreated cells preserved their viability throughout the 54 min period, declining slightly to 97 ± 1% for both strains. The x-axis represents the number of viable cells present (expressed as a percentage) after corresponding EMF exposures and the y-axis represents the number of EMF exposures.

Due to the lack of data on the effect the multiple 18 GHz EMF exposures has on bacterial cells, two *Staphylococcus aureus* strains were subjected to 18 treatments over a one-hour period to monitor the permeability of the cells, together with the number of viable cells grown on the nutrient agar (NA) plates. The CLSM analysis of EMF exposed cells showed that after the second exposure, the cells of both strains had become permeable (up to 99% of the treated cells, p < 0.05; S4 Fig) and remained so for the remainder of the EMF exposures. As the number of EMF exposures were increased up to the seventh and eighth exposure, a gradual decline in bacterial cells recovered on nutrient agar plates was observed, decreasing to 54 ± 5% (p < 0.05) for *Staphylococcus aureus* ATCC 25923 (8^th^ exposure) and 46 ± 5% (p < 0.05) for *Staphylococcus aureus* CIP 65.8^T^ cells (7^th^ exposure). After subsequent exposures, however, the cell number was found to increase periodically, up to 78 ± 5% (p < 0.05) for *Staphylococcus aureus* ATCC 25923 (9^th^ exposure) and 60 ± 5% (p < 0.05) for *Staphylococcus aureus* CIP 65.8^T^ (8^th^ exposure). Hence, despite the overall decline in the viable cell numbers over continuous EMF exposures, it is noteworthy that after the seventh/eighth, thirteenth/fourteenth, and sixteenth/seventeenth EMF exposures, the number of cells recovered on the plates was found to increase for both strains (p < 0.05, Fig 6).

The periodic increases in cell number (inferred from the direct counting of viable cells) throughout the multiple 18 GHz EMF exposures is a notable phenomenon which, to the best our knowledge, has not been previously reported. Copty *et al*. suggested that the enzyme kinetics could be changed by heating the water molecules that are attached to the cellular proteins [60]. This perhaps may attribute to the increases in cell number.

## Conclusion

A fundamental understanding of the phenomenon of permeabilisation of prokaryotic and yeast cells by exposing them to 18 GHz EMF radiation is important. The results of this study have demonstrated that exposure to 18 GHz EMF induced cell permeability in all of the organisms being studied in a manner that could not be duplicated using conventional heating methods under similar temperature conditions, regardless of the differences in cell wall/ membrane structures of each type of cell. It was established that the threshold level of EMF exposure that was required to induce cell permeability to allow the passage of large nanospheres (46 nm) through the cell membrane was in the range of three and six EMF exposure doses with specific absorption rate (SAR) of 3 kW/kg and 5 kW/kg per exposure, depending on the type of cell.

In addition, it was found that multiple 18 GHz EMF exposures could induce notable periodic increases in the cell number of *Staphylococcus aureus* CIP 65.8^T^ and ATCC 25923. Further study of the effect of EMF exposure has on multicellular eukaryotes and liposomes (with or without proteins) is required in order to fully evaluate the effects of such multiple EMF exposures have on biological systems and its applicability as a new drug delivery/release technique due to its non-invasive nature, local application, and cost-effectiveness.

## Supporting Information

S1 Fig No uptake of 23.5 nm nanospheres by the control groups. Typical TEM images of thin-sectioned (80 nm) cells showing 23.5 nm nanospheres outside and around the cell membrane of untreated and heat treated cells and uniform cytosol with no nanospheres. Scale bars are 200 nm.
(TIF)

S2 Fig Cells response to Peltier heat treatment and untreated cells. SEM, CLSM and phase contrast images showing unchanged cell appearance with no nanosphere intake. Scale bars 1 μm (top row) and 5 μm (second and third rows).
(TIF)

S3 Fig No propidium iodide uptake by the control groups. CLSM images showing no propidium iodide internalization after EMF exposure (first row). Phase contrast micrographs showing cells in the same field of view (second row). Scale bars in all fluorescence images are 5 μm.
(TIF)

S4 Fig The effect of multiple 18 GHz EMF exposures on the morphology and permeability of *Staphylococcus aureus* cells. Typical scanning electron micrographs of *S*. *aureus* ATCC 25923 and *S*. *aureus* CIP 65.8^T^ cells after multiple 18 GHz EMF exposures. No significant change in cell morphology was observed up to the 7^th^ exposure (insets). Scale bars are 10 μm, inset scale bars are 200 nm. CLSM images showing intake of 23.5 nm nanospheres (second and fifth row) after the 2^nd^ exposure. The phase contrast images in the bottom row show the bacterial cells in the same field of view. Scale bars are 5 μm.
(TIF)

S1 Table Phospholipids compositions of cell membranes in 18 GHz EMF exposure studies. (DOCX)
