# Supplementary figures and images for: The Bioeffects Resulting from Prokaryotic Cells and Yeast Being Exposed to an 18 GHz Electromagnetic Field
Source: PLoS One. 2016 Jul 8;11(7):e0158135. doi: 10.1371/journal.pone.0158135 (PMC4938218; doi:10.1371/journal.pone.0158135)

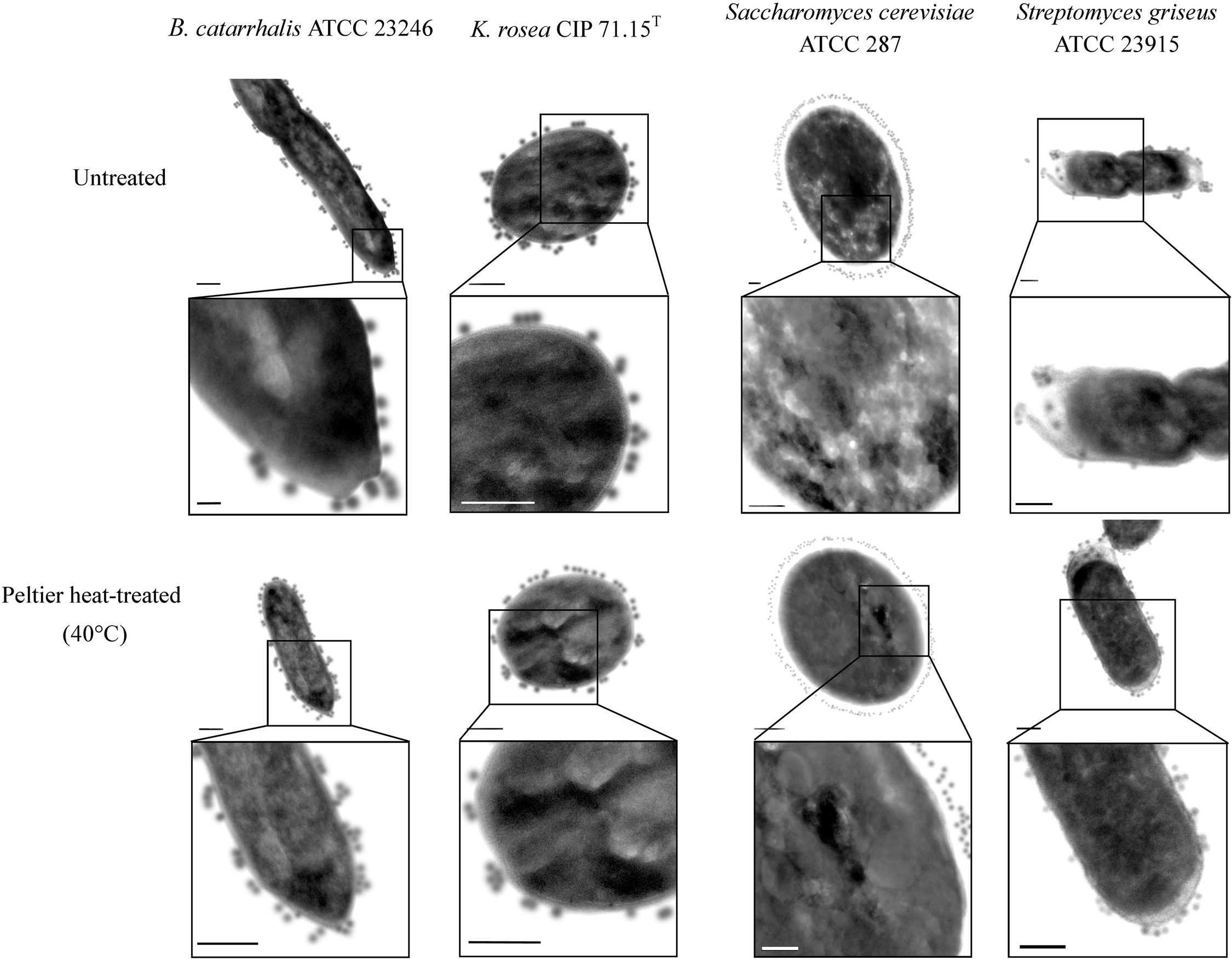

Supplement: S1 Fig — Typical TEM images of thin-sectioned (80 nm) cells showing 23.5 nm nanospheres outside and around the cell membrane of untreated and heat treated cells and uniform cytosol with no nanospheres. Scale bars are 200 nm. (TIF) [file pone.0158135.s001.tif]

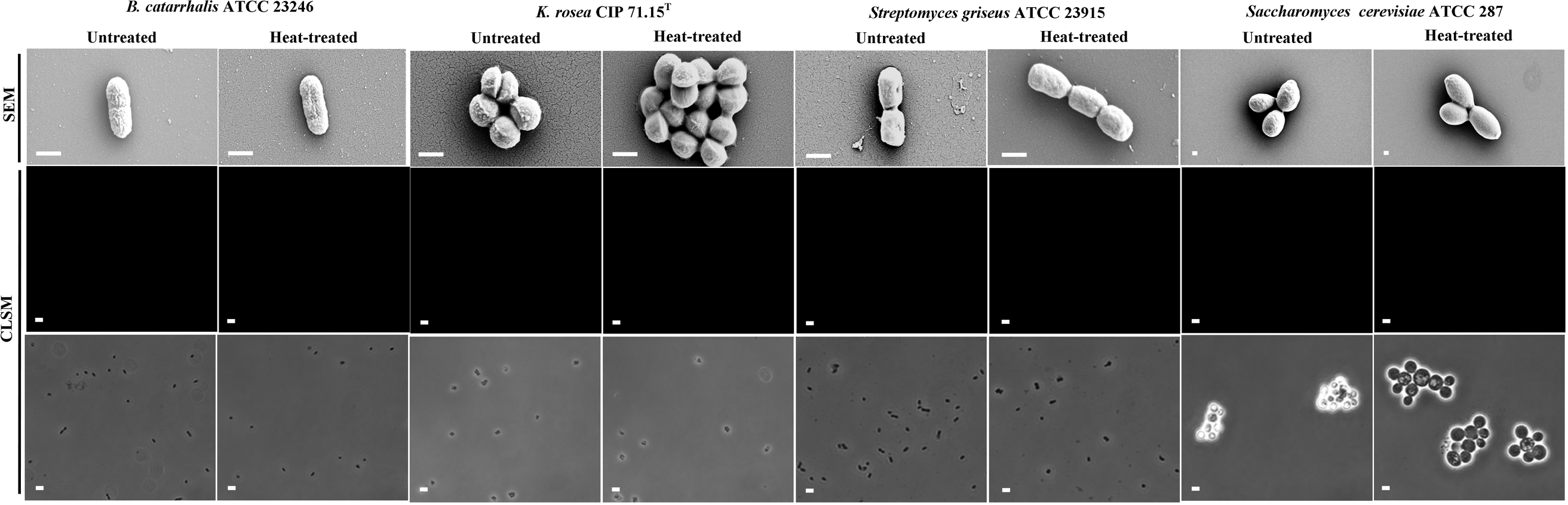

Supplement: S2 Fig — SEM, CLSM and phase contrast images showing unchanged cell appearance with no nanosphere intake. Scale bars 1 μm (top row) and 5 μm (second and third rows). (TIF) [file pone.0158135.s002.tif]

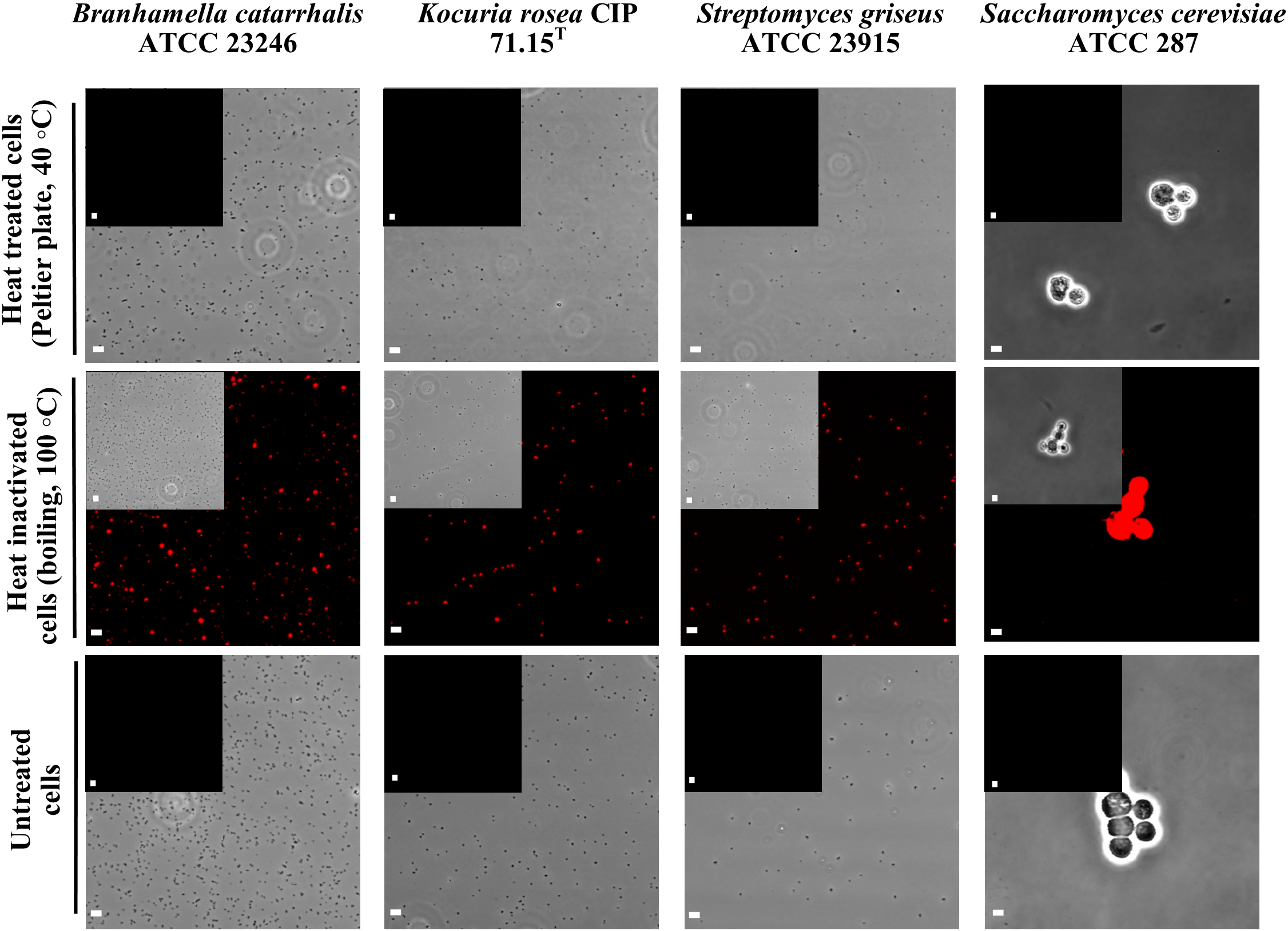

Supplement: S3 Fig — CLSM images showing no propidium iodide internalization after EMF exposure (first row). Phase contrast micrographs showing cells in the same field of view (second row). Scale bars in all fluorescence images are 5 μm. (TIF) [file pone.0158135.s003.tif]

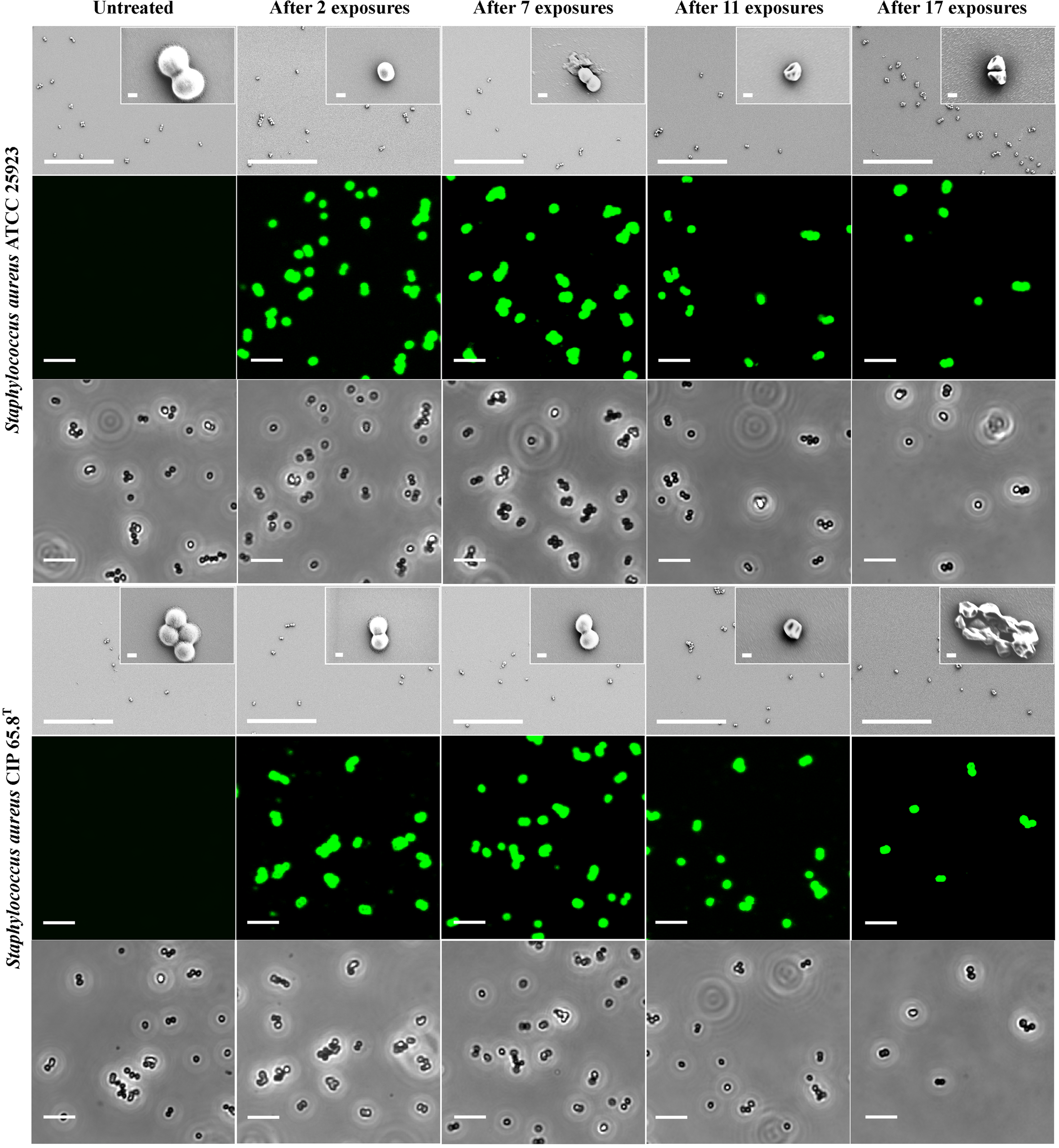

Supplement: S4 Fig — Typical scanning electron micrographs of S. aureus ATCC 25923 and S. aureus CIP 65.8T cells after multiple 18 GHz EMF exposures. No significant change in cell morphology was observed up to the 7th exposure (insets). Scale bars are 10 μm, inset scale bars are 200 nm. CLSM images showing intake of 23.5 nm nanospheres (second and fifth row) after the 2nd exposure. The phase contrast images in the bottom row show the bacterial cells in the same field of view. Scale bars are 5 μm. (TIF) [file pone.0158135.s004.tif]
