# Supplementary material for: The Bioeffects Resulting from Prokaryotic Cells and Yeast Being Exposed to an 18 GHz Electromagnetic Field
Source: PLoS One. 2016 Jul 8;11(7):e0158135. doi: 10.1371/journal.pone.0158135 (PMC4938218; doi:10.1371/journal.pone.0158135)
Supplement: S1 Table — (DOCX) [file pone.0158135.s005.docx]

**S1 Table. Phospholipids compositions of cell membranes in 18 GHz EMF exposure studies.**

|  | Phospholipids | | | | | | | |
| --- | --- | --- | --- | --- | --- | --- | --- | --- |
| Species | Phosphatidic acid (PA) | Phosphatidyl-choline (PC) | Phosphatidyl-ethanolamine (PE) | Phosphatidyl-glycerols (PG) | Diphosphatidyl-glycerol (DPG) | Lysyl-PG | Phosphatidyl-inositol (PI) | Others |
| *Branhamella catarrhalis*[1] | 0 | 3 | 38 | 41 | 18 | 0 | 0 | 0 |
| *Escherichia coli*[2] | 0 | 0 | 91 | 3 | 6 | 0 | 0 | 0 |
| *Kocuria rosea*[3] | 0 | 0 | 0 | 74 | 26 | 0 | 0 | 0 |
| *Planococcus maritimus*[4, 5] | 0 | 0 | 8-14 | 23-38 | 44-45 | 0 | 0 | 0 |
| *Staphyloccoccus aureus*[6] | 0 | 0 | 0 | 57 | 5 | 38 | 0 | Traces |
| *Staphyloccoccus epidermidis*[7] | 0 | 0 | 0 | 90 | 1 | 0 | 0 | 9 |
| *Streptomyces griseus*[8] | 0 | 0 | + | 0 | + | 0 | + | 0 |
| *Saccharomyces cerevisiae*[9] | 2 | 35 | 24 | 0 | 0 | 0 | 35 | 0 |

**References**

1. Beebe JL, Wlodkowski TJ. Lipids of *Branhamella catarrhalis* and *Neisseria gonorrhoeae*. J Bacteriol. 1976;127(1):168-78.

2. Lugtenberg EJJ, Peters R. Distribution of lipids in cytoplasmic and outer membranes of Escherichia coli K12. Biochim Biophys Acta. 1976;441(1):38-47.

3. Whiteside TL, De Siervo AJ, Salton MR. Use of antibody to membrane adenosine triphosphatase in the study of bacterial relationships. J Bacteriol. 1971;105(3):957-67.

4. Thirkell D, Summerfield M. The effect of varying sea salt concentration in the growth medium on the chemical composition of a purified membrane fraction from *Planococcus citreus* Migula. Anton Leeuw Int J G. 1977;43(1):37-42.

5. Miller KJ. Effects of temperature and sodium chloride concentration on the phospholipid and fatty acid compositions of a halotolerant Planococcus sp. J Bacteriol. 1985;162(1):263-70.

6. Haest CWM, De Gier J, Op Den Kamp JAF, Bartels P, Van Deenen LLM. Changes in permeability of *Staphylococcus aureus* and derived liposomes with varying lipid composition. BBA - Biomembranes. 1972;255(3):720-33.

7. Komaratat P, Kates M. The lipid composition of a halotolerant species of *Staphylococcus epidermidis*. Biochim Biophys Acta. 1975;398(3):464-84.

8. Lechevalier MP. Lipids in bacterial taxonomy - a taxonomist's view. CRC Crit Rev Microbiol. 1977;5(2):109-210. Epub 1977/01/01.

9. Rank GH, Robertson AJ. The viscosity and lipid composition of the plasma membrane of multiple drug resistant and sensitive yeast strains. Can J Biochem. 1978;56(11):1036-41.
